# Efficacy and safety of bilateral same session flexible ureteroscopy using flexible and navigable suction ureteral access sheath (FANS) versus conventional access sheath: a prospective randomized comparative study

**DOI:** 10.1007/s00345-025-06126-x

**Published:** 2025-12-25

**Authors:** Helmy Eldib, Moaz Abdelrahman, Islam Nouh, Mahmoud Mobarak, Mahmoud Farag

**Affiliations:** Urology Department, Benha UniversityBenha University, Benha, Egypt

**Keywords:** Novel, UAS, Flexible URS, Traditional, Bilateral, Simultaneous

## Abstract

**Aim:**

While research on the comparison between the Flexible and Navigable Suction Ureteral Access Sheath (FANS) and the traditional ureteral access sheath (TUAS) in flexible ureteroscopy for unilateral stones is scarce, this study aims to assess their performance during bilateral same-session flexible ureteroscopy, examining safety, efficacy, and perioperative outcomes.

**Patients and methods:**

In total, 100 cases aged 23 to 65 with bilateral stones under 20 mm were randomized into two groups: one using the NUAS and the other the TUAS. Exclusion criteria encompassed pediatric cases, individuals with untreated coagulopathies, and those with stones in calyceal diverticula.

**Results:**

A significant difference was observed in the distribution of stone-free rates (SFRs) between the groups, with the FANS group demonstrating a elevated grade A (absolute stone-free) rate and fewer grade C (2.1 to 4 mm residual stones) as opposed to the TUAS one (70% vs. 20%, and 6% vs. 48%, *p* < 0.001). Additionally, the operative time was significantly longer in the TUAS group (58.4 ± 14.53 min) as opposed to the FANS group (48.41 ± 13.9 min, *p* = 0.001). The infection rate was also significantly elevated in the TUAS group (16%) as opposed to the FANS group (4%, *p* = 0.046). In terms of intraoperative and postoperative adverse events, the groups were similar (*p* < 0.05).

**Conclusion:**

FANS emerges as a promising and safer approach for bilateral flexible ureteroscopy, offering superior SFRs, faster procedure times, and fewer infections when as opposed to the traditional TUAS approach.

## Introduction

The prevalence of newly diagnosed bilateral stone disease has seen a marked increase, with approximately 15% of cases experiencing simultaneous involvement of both renal units [1].

As a result, a variety of surgical treatment options have emerged for managing bilateral stone disease, including shock wave lithotripsy, percutaneous nephrolithotomy (PCNL), flexible ureteroscopy, or a combination of these approaches [2].

Advancements in the technology behind flexible ureteroscopy have significantly enhanced its superiority over traditional methods like PCNL and shock wave therapy. This is especially true in cases that involve coagulopathy, congenital renal malformations, and excessive obesity. Flexible ureteroscopy has proven to offer several notable advantages as opposed to PCNL, such as reduced trauma, a significantly diminished incidence of adverse events (AEs), and a markedly shorter postoperative hospital stay, making it an increasingly preferred choice for many clinicians [3 and 4].

In particular, when bilateral flexible ureteroscopy is performed simultaneously, the procedure has the potential to drastically reduce overall operative time, minimize anesthetic requirements, and shorten both the length of hospital stay and the recovery period, when as opposed to staged procedures. This makes simultaneous flexible ureteroscopy not only an effective but also a more efficient option for cases [5].

However, despite these benefits, many surgeons remain apprehensive about the potential elevated risk for intraoperative AEs, especially the possibility of injuring both ureters, which could lead to significant long-term morbidity and AEs [6].

The conventional flexible URS access sheath, while commonly used, has some inherent limitations. One such limitation is the persistence of stone fragments or dust, which can extend the duration of the procedure and decrease the overall efficacy of lithotripsy. This issue, in turn, increases the likelihood of AEs, particularly in the context of simultaneous bilateral flexible URS procedures [7].

However, with the continuous advancements in endoscopic technology, a flexible and Navigable Suction Ureteral Access Sheath (FANS) has been developed, which has shown promising results in clinical practice. This newly designed sheath has demonstrated improved postoperative outcomes, including enhanced efficacy and significantly elevated stone-free rates (SFRs) [8].

Therefore, we compare between novel access sheath and traditional access sheath in simultaneous bilateral flexible ureteroscopy.

## Patient and methods

### Study design

A prospective, randomized study was conducted at Benha University Hospitals involving adult cases diagnosed with renal and/or upper ureteric stones who met specific inclusion criteria. These participants were enrolled in a comparative study evaluating the efficacy of a flexible and Navigable Suction Ureteral Access Sheath (FANS) versus the TUAS, all performed during the same-session bilateral flexible ureteroscopy. This study was conducted according to ethical principles stated in the Declaration of Helsinki (2013) [9] and the requirement of faculty of medicine, Benha university.

Following the completion of the informed consent process, cases were randomly allocated into one of two groups using the closed-envelope method. Both patients and surgeons were aware of the type of surgery after opening the envelope. Only external assessors were blinded “assessor blinded”.

**Group A**: This group consisted of 60 cases who underwent surgery utilizing the TUAS during flexible ureteroscopy.

**Group B**: Similarly, 60 cases in this group underwent surgery using the FANS during flexible ureteroscopy.

### Inclusion and exclusion criteria

- *Inclusion Criteria*: Adults with bilateral upper tract stones measuring less than 20 mm.
- *Exclusion Criteria*: Pediatrics with untreated coagulopathies, or stones located in a calyceal diverticulum.

Detailed demographic information—including age, sex, body mass index (BMI), and pre-existing comorbidities—was systematically collected for all participants. Each patient also underwent a comprehensive assessment comprising a thorough medical and surgical history, physical examination, and urine culture to rule out active urinary tract infections. In case of positive urine culture, the patient was given the proper antibiotic according to urine sensitivity test. Furthermore, non-contrast computed tomography (NCCT) scans of the abdomen and pelvis (Fig. 2) were performed for every individual to evaluate stone characteristics, including location, size, number, the presence and degree of hydronephrosis, and Hounsfield unit measurements.

During the procedure, we would normally treat the symptomatic side first and in case of bilateral same symptoms it was according to surgeon preference to begin with the more difficult one, in terms of larger stone burden and difficult access to the stone, or to begin with the easier side with less stone burden and easier access to the stone.

In both groups,

All pertinent perioperative parameters were meticulously recorded for subsequent analysis. All patients were assessed 1 month post-operatively by NCCT to assess their stone free rate and they were categorized as follows: Stone free rate Grade A: absolute free, Grade B: (≤ 2 mm) & Grade c: (2.1 to 4 mm).

### Approach

#### Group A: flexible ureteroscopy with TUAS

**Fig. 1:**
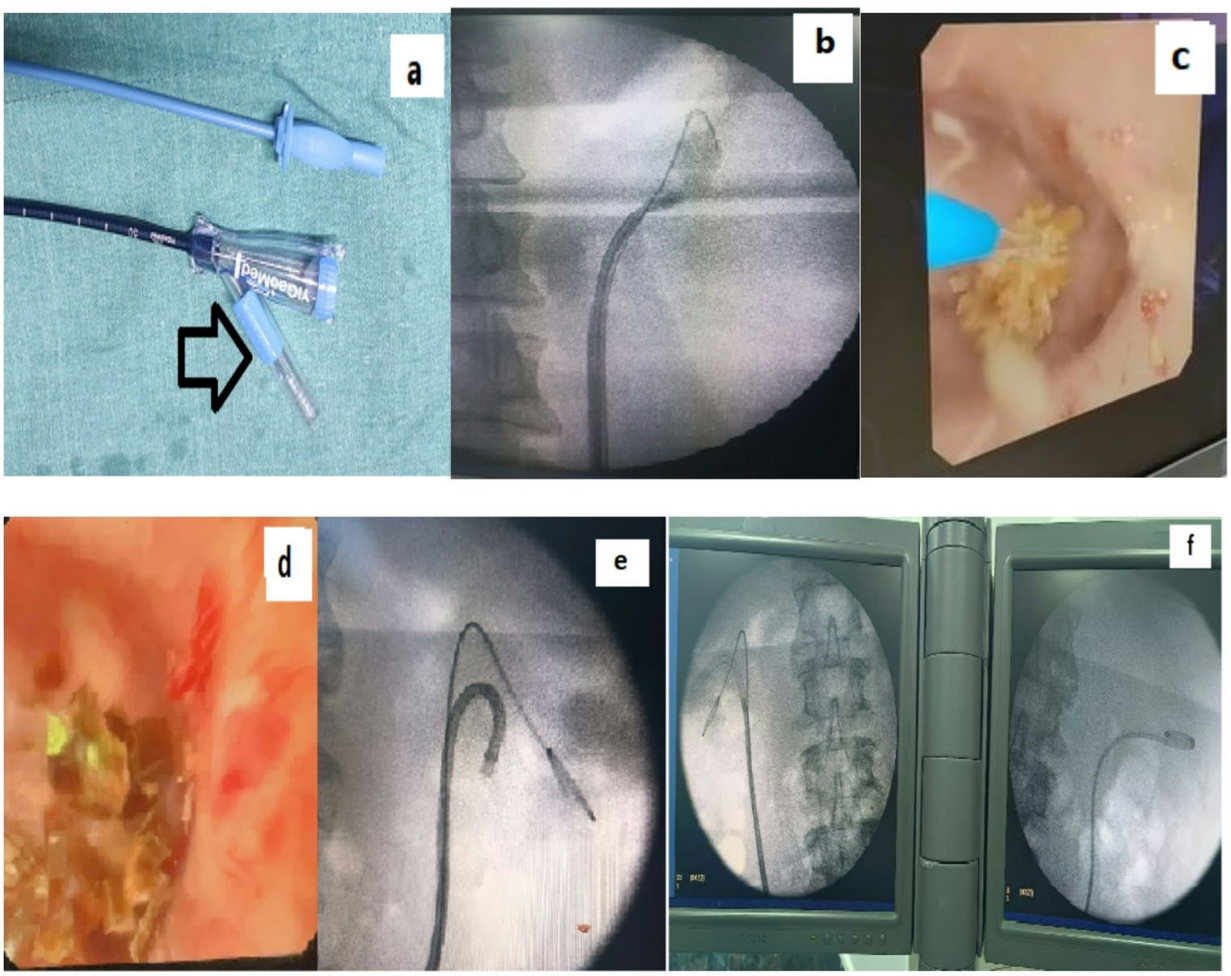
**a** Novel ureteral access sheath versus traditional access sheath " black arrow showing suction part of novel access sheath (**b**): Traditional ureteral access sheath over safety guide wire with noted gap between it and the stone to allow bending of flexible URS. (**c**) Stone fragmentation with laser probe in contact with the stone in a calyx (d) Fragmented stone in one of kidney calyces. (**e**) Novel Flexible ureteral access sheath (f-UAS) with a 10 cm front end that bends passively in conjunction with the f-URS. (**f**) Bilateral case with left JJ after procedure completion and right flexible URS

Under general anesthesia (GA) and with appropriate antibiotic prophylaxis, cases were positioned in the lithotomy position. Initial access was gained via rigid cystoscopy to identify the ureteric orifice, through which a safety guidewire was inserted. A semi-rigid ureteroscope was then advanced over a secondary working guidewire up to the pelvi-ureteric junction (PUJ) or the most proximal reachable segment, serving to facilitate passive ureteric dilation. In certain instances, active dilation with sequential dilators (up to 14 French) was performed. Two calibers of traditional ureteral access sheaths (UAS) from COOK Medical—Flexor 12/14 Fr (larger) and 9.5/11.5 Fr (smaller)—were employed based on the extent of dilation required. The selected sheath was advanced over the guidewire and positioned just distal to the PUJ or directly below the upper ureteral stone under fluoroscopic guidance (Fig. 1). Flexible ureteroscopy was then performed using an 8.5 Fr Olympus URF-V2 ureteroscope, permitting thorough inspection of the proximal ureter for stone detection. Lithotripsy was carried out utilizing a Holmium: YAG laser (20 W; Lumenis, UK) operating at an energy setting of 1.0–1.2 joules and a frequency of 15–30 Hz, equipped with a 272-micron fiber. When stone extraction was necessary, a retrieval basket was employed (Fig. 1).

#### Group B: flexible ureteroscopy with FANS

**Fig. 2:**
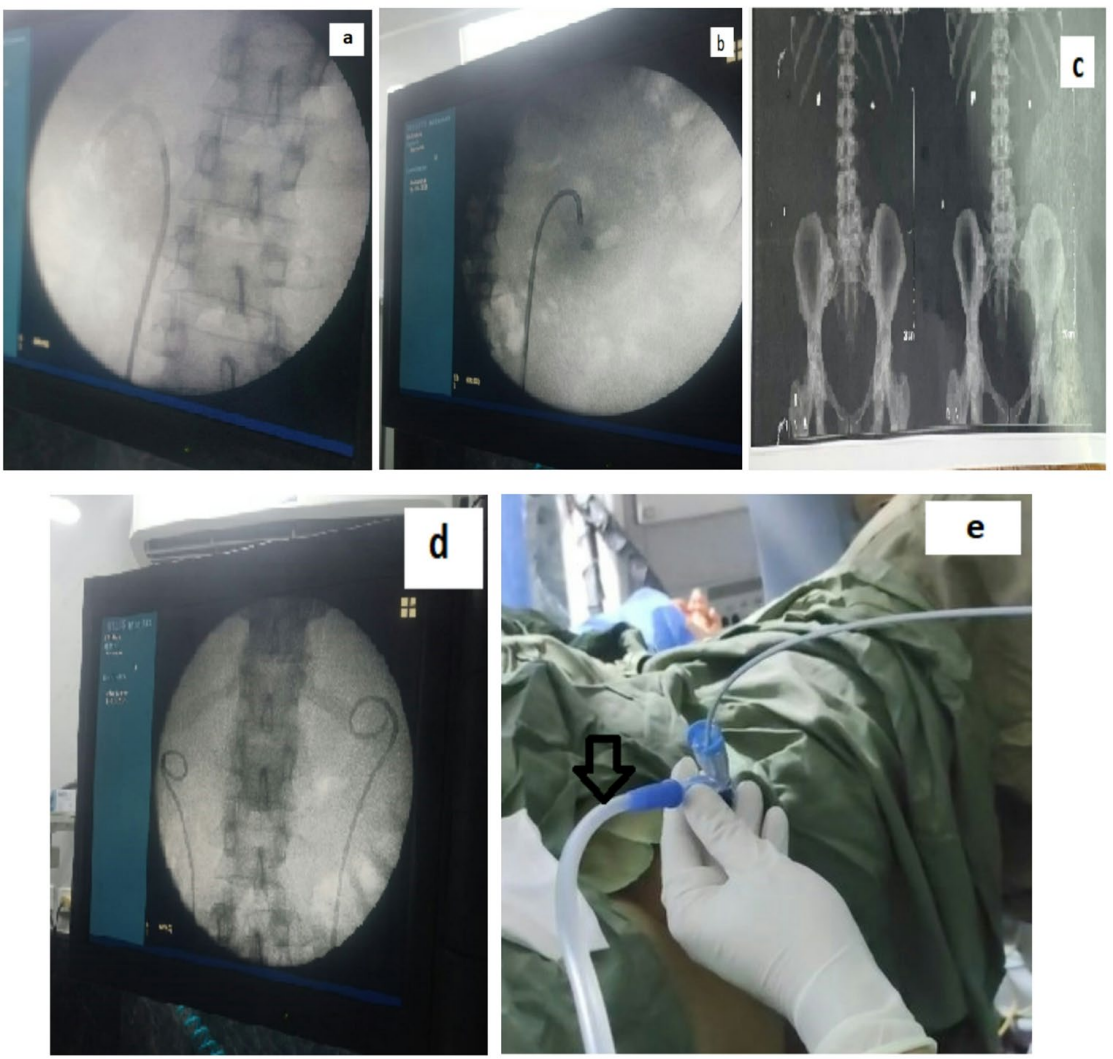
(**a**): flexible URS going through bended tip of Novel ureteral access sheath (**b**): bended tip of novel access sheath in close proximity to the stone. (**c**) CT” KUB " showing bilateral renal stone (**d**) Fluoroscopic image of a stone free case with bilateral jj after flexible URS using novel access sheath. (**e**) Suction port of novel access sheath connected to vacuum suction device

In this cohort, a Flexible and Navigable Suction Ureteral Access Sheath (FANS) was employed (Fig. 1). This device is distinguished by a 10 cm distal segment that passively follows the deflection of the f-URS, enabling a remarkable bending angle of up to 270° while preserving a cylindrical lumen configuration during flexion. A notable feature of this innovative f-UAS is its integrated capability for vacuum suction via connection to a negative pressure device (Fig. 2).

Following the induction of GA, cases were positioned in the lithotomy posture, with individual adjustments such as Trendelenburg (head-low), reverse Trendelenburg (foot-high), or lateral tilting, depending on case-specific anatomical and procedural considerations. After identification of the ureteric orifice and placement of a safety guidewire, a 9.8 Fr semi-rigid ureteroscope (Karl Storz, Germany) was utilized for initial ureteral assessment. In instances where upper ureteral calculi were encountered, stones were retrogradely relocated into the renal pelvis.

Subsequently, the FANS was advanced over the guidewire, with its distal tip positioned in close proximity to the target stone within the renal pelvis or calyceal system under direct vision via an 8.6 Fr f-URS (Fig. 2).We used two sizes of FANS, 10&12 fr (Brand Yigao Med, elephant ll) according to the degree of ureter dilatation. Once appropriately situated, the FANS was connected to a vacuum suction system, and a negative pressure of 2–7 kPa was applied. This pressure was modulated intraoperatively through a vent control, tailored to procedural dynamics. Continuous irrigation was maintained at a flow rate of 90–200 mL/min to ensure adequate visualization and cooling.

Stone fragmentation was performed using a Holmium: YAG laser (20 W; Lumenis, UK) equipped with a 200 μm fiber, operating at an energy setting of 1.0–1.2 joules and a frequency of 15–30 Hz. Fragment evacuation was facilitated by leveraging the irrigation outflow and active suction, with repeated withdrawal of the ureteroscope to flush out disintegrated stone material effectively.

## Results

Table 1 shows preoperative data of the study groups. This study was conducted on 100 patients divided into two equal groups, TUAS group and FANS group (50 patients in each group). Age ranged from 23.0 to 72.0 years. Males consisted more than half of study groups, 58% of TUAS group and 56% of FANS group. There was no significant difference in distribution of age and sex between study groups, *p* = 0.976 and 0.840, respectively.

Regarding medical and surgical history, 86% of patients in the study were free from comorbidities, 66% of patients had no previous surgical history, with no statistical difference between groups, *p* < 0.05.

There was no significant difference between two groups in stone characters (RT and left stone size, location and Hounsfield unite), *p* < 0.05.

Majority of patients (87%) suffered from loin pain, only 26% of patients complained hematuria. Normal count of pus cells in urine analysis was found in 68% of TUAS group versus 72% of FANS group, with no significant difference, *p* < 0.05. Positive urine cultures were found in 30% of TUAS group versus 24% of FANS group, *p* = 0.499.

Considering kidney function test, there was no significant difference in serum creatinine level between both groups, *p* = 0.586. There was no significant difference between groups in main complaint nor urine analysis.

**Table 1:** Preoperative data of the study groups

Variable	TUAS group (*n* = 50)			FANS group (*n* = 50)		*P* value
Demographics						
Age (Years) Mean ± SD Range	49.42 ± 9.32 23.0–71.0			49.36 ± 10.09 24.0–72.0		0.976
Sex Male Female	*N*	*%*		*N*	*%*	0.840
	29 21	58.0 42.0		28 22	56.0 44.0	
History of surgery Free PCNL Pyelolithotomy URS or SWL	31 2 2 15	62 4 4 30		35 1 2 12	70 2 4 24	0.823
Stone Description						
Stone Side Bilateral	50	50		50	50	----
RT Stone Size (mm) Mean ± SD Range	15.9 ± 2.121 11.0–19.0			15.72 ± 2.10 12.0–19.0		0.671
LT Stone Size (mm) Mean ± SD Range	15.55 ± 1.962 11.0–20.0			15.5 ± 2.043 12.0–19.0		0.901
RT Stone Location Pelvis Lower calyx Middle calyx Upper calyx	34 2 7 7	68 4 14 14		32 2 8 8	64 4 16 16	0.966
LT Stone Location Pelvis Lower calyx Middle calyx Upper calyx	21 15 4 10	42 30 8 20		26 10 3 11	52 20 6 22	0.630
RT Hounsfield unite Mean ± SD Range	957.7 ± 322.4 450.0-1700.0			980.9 ± 313.4 100.0-1800.0		0.720
LT Hounsfield unite Mean ± SD Range	1014.3 ± 254.7 550.0-1650.0			1012.7 ± 189.6 650.0-1550.0		0.972
Kidney function and urine analysis						
Serum creatinine (mg/dl) Mean ± SD Range	1.029 ± 0.282 0.5–1.9			1.00 ± 0.239 0.5–1.8		0.586
Pus cells 0–10 -50 -100 - over 100	34 10 4 2		68 20 8 4	36 9 4 1	72 18 8 2	0.931
Mid-stream positive culture	15		30	12	24	0.499
Urine culture (*n* = 27) E coli Klebsiela Other organisms	11 2 2		73.4 13.3 13.3	9 2 1	75.0 16.7 8.3	0.885

Data are expressed as mean ± SD, Range (Min-Max), number and percentages, used tests of significance: Independent T test, Chi square and Fisher Exact tests, significance level at *P* < 0.05

There was no significant difference between two groups in intra-operative complications (mucosal injury, bleeding, failed operation, perforation, false passage and conversion to other procedure), *p* < 0.05. On the other hand, there was highly significant increase in operative time (58.4 ± 14.53 in TUAS group versus 48.41 ± 13.9 in TUAS group, *p* = 0.001*).

There was no significant difference between two groups in post-operative complications (fever, pain, hematuria, other complications, readmission and stent duration), but there was significant difference in infection between the groups (18% with TUAS vs. 4% with FANS, *p* = 0.025).

There was significant difference in distribution of stone free rate between both groups as FANS group showed higher grade A (absolute free rate) and minimal grade C (2.1 to 4 mm stone residuals) compared to TUAS group, (70% versus 20%, and 6% versus 48%), *p* < 0.001*).

Significant difference was found in ureteral post-operative stenting between groups (58% with TUAS vs. 34% with FANS, *p* = 0.016*), but there was no significant difference between both groups in pre-operative stenting and stenting time (Tables 2 and 3).

**Table 2:** Distribution of intra-operative and post-operative data in study groups:

Variable	TUAS group (*n* = 50)		FANS group (*n* = 50)		*P* value
	*N*	%	*N*	%	
Intra-operative data					
Operative time (min) Mean ± SD Range	58.4 ± 14.53 19.3–76.3		48.41 ± 13.9 19.8–69.7		0.001**
Intra-operative complications					
Mucosal injury	4	8	2	4	0.400
Bleeding	9	18	5	10	0.249
Failed*	2	4	1	2	0.558
False passage	3	6	1	2	0.307
Converted to other operation	3	6	0	0	0.242
Post-operative data					
Infection	9	18	2	4	0.025*
Fever	7	14	2	4	0.081
Pain	12	24	9	18	0.461
Hematuria	15	30	11	22	0.362
Readmission	2	4	0	0	0.459
Other Complications	3	6	0	0	0.242
Stone free rate Grade A: absolute free Grade B: (≤ 2 mm) Grade c: (2.1 to 4 mm)	10 16 24	20 32 48	35 12 3	70 24 6	< 0.001**
Stenting					
Pre-operative ureteral stenting	11	22	13	26	0.640
Post-operative stenting	29	58	17	34	0.016*
Stent duration(days) Mean ± SD Range	24.48 ± 5.195 15.90–30.40		25.48 ± 4.87 15.2–39.9		0.323

Data are expressed as mean ± SD, Range (Min-Max), number and percentages, used tests of significance: chi square, Fisher Exact and independent t tests, *: significant at *P* < 0.05, **: Highly significant at *P* < 0.01

* Failed: include cases of failed procedure on one side due to encountered ureteric stricture, active haematuria obscuring surgical field & adverse events leading to ending procedure on that side, stenting and intervention later on

Univariate binary logistic regression analysis was performed to weigh the risk of different variables as predictors of operative time, bleeding, stone free rate and post-operative urinary tract infection. Technique used was significant predictor for operative time and stone free rate; presence TUAS technique increases the risk of increasing operative time by 8.757 folds more than FANS technique. TUAS technique increased the risk of bleeding by 3.917 folds more than FANS technique. FANS technique significantly increased the odds of free stone rate of grade A by 8.415 times more than other technique (TUAS), *p* < 0.001*.

Regarding post-operative infection, stone size, pre-operative positive culture, technique used and operative time were all significant predictors. Each one unit increased in stone size increases the risk of infection by 1.740 times. Pre-operative positive culture increased the risk of infection by 8.377 times. FANS reduced the risk of infection by 81% more than TUAS technique. Each one unit increased in operative time increases the risk of infection by 1.052 times.

**Table 3:** Logistic regression analysis for the different variables as risk factors of post-operative urinary tract infection:

Risk Factors	Univariate Analysis		Multivariate Analysis			
	OR (95% C.I)	Sig.	OR (95% C.I)		Sig.	
Age (Years)	1.010(0.944–1.088)	0.774				
Gender, male	1.705(0.414–7.024)	0.460				
Stone size	1.567(1.072–2.291)	0.020*	0.012*	1.740(1.132–2.675)		
Positive Urine culture	4.421(1.216–16.070)	0.024*	0.012*	8.377(1.590-44.127)		
Upper Ureter	1.481(0.282–7.766)	0.642				
Middle Ureter	0.604(0.071–5.143)	0.644				
Lower Ureter	1.481(0.282–7.766)	0.642				
Pelvis	1.226(0.269–5.075)	0.779				
Preoperative stent	0.762(0.201–2.884 )	0.063				
Technique, UAS	6.127(1.186–31.651)	0.030*	0.040*		0.190(0.0.039–0.929)	
Operative time	1.186(1.020–1.212)	0.016*	0.035*		1.052(1.009–1.086)	
Constant			< 0.001*		0.096	
Model significance			0.034*			
Classification percentage			89%			
Pseudo r square			0.119			

OR: odds ratio, 95%(C.I.): 95% confidence interval, sig.: significant at *P* < 0.05

## Discussion

In the last decade, bilateral simultaneous flexible URS has gained popularity over unilateral flexible URS as it provides similar outcomes in terms of SFR and incidence of ureteral AEs [10, 11].

Additionally, bilateral same session flexible URS is associated with less exposure to anesthesia, more rapid recovery, less number of required hospitalizations and reduced overall operative room time [12, 13].

The employment of FANS in bilateral same session cases, yields even more better outcomes with its tip characters allowing more efficacious stone fragmentation and its suction part that provides more clear vision through flushing of small stone fragments decreasing operative time and its associated AEs with better SFR [8].

In our research, we observed a significant difference in the distribution of stone-free rates between the two groups, with the FANS group demonstrating a elevated percentage of grade A (complete stone-free) outcomes and a significantly diminished occurrence of grade C (2.1 to 4 mm stone residuals) as opposed to the TUAS one, (70% versus 20%, and 6% versus 48%, respectively, *p* < 0.001*). This discrepancy can be explained by the employment of FANS in flexible URS with its suction part and its tip characters is associated with less residual stone fragments that can lead to renal colic, hematuria, the need for retreatment, and forming a nucleus for stone regrowth. Additionally, the suction part helps controlling intra-renal pressure and obtaining a clear surgical field yielding better stone free rate [13].

Consistently, Zhang and co-authors 2023 [8] reported that the FANS use allows the flexible URS to be as close as possible to the stone, therefore, the gap between the f-URS and the 12/14 Fr f-UAS creates an ideal environment for flushing out stone fragments ≤ 1 mm, while larger fragments, ranging from 1 to 4 mm, are efficiently cleared via the rhythmic in-and-out movement of the f-URS. This dynamic motion, coupled with the outflow of irrigation fluid during the f-URS withdrawal, is further amplified by the suction mechanism within the sheath, ensuring a thorough and effective expulsion of stone debris. All these factors contribute to the novel access sheath superiority over traditional access sheath in improving stone free rate [8].

On the same line, Gauhar and co-authors 2025 [14] gathered data from 115 adults with bilateral renal stones, who were managed with bilateral simultaneous flexible URS using Flexible and Navigable Suction Ureteral Access Sheath (FANS) across 14 centers worldwide. The study reported that, at 30 days post-procedure, only 2 cases (1.7%) had residual fragments larger than 4 mm, while only 2.65% of the cases required a planned re-intervention [15].

Other researchers compared the outcomes of FANS versus traditional ureteral access sheath and the novel-UAS group demonstrated significantly elevated SFRs, with 86.3% on day 1 and 91.2% at 30 days, outperforming those treated with TUAS despite having similar stone burdens ( [15, 16]& [17]).

Which agrees with Yu and co-authors 2024 who reported a SFR of 76.3% in the FANS group as opposed to 7.2% in the TUAS group (*P* < 0.001) on postoperative day 1. They believed that elevated SFR in the novel-UAS group contributes to reduced incidence of renal colic or hematuria that can be caused by residual stone fragments and Zhu and co-authors 2019 [19] who stated that, when comparing it to traditional-UAS, suction part added to FANS can enhance stone free rate(20& 21).

The present investigation revealed a significant difference in infection rates between the two groups, with 18% of cases in the TUAS group and only 4% in the FANS group experiencing infections (*p* = 0.025). The elevated infection rate in the traditional group is likely due to the rigid tip of the conventional UAS, which must be positioned at the UPJ, where it can become obstructed by the ureteral mucosa and lead to elevated intra-renal pressure. In contrast, the FANS offers enhanced flexibility, allowing for smoother navigation through the UPJ, which reduces mucosal injury and the AEs associated with higher intra-renal pressure [20].

Elevated intra-renal pressure associated with flexible ureteroscopy can lead to intraoperative fluid absorption (pyelo-venous and pyelo-lymphatic backflow), extravasation leading to postoperative fever and infectious AEs up to urosepsis [21].

On the other hand, the flexible tip of the FANS with about 270°passive bending capability helps maneuvering it easily together with f-URS across the UPJ into the renal pelvis or calyces. The wide space through which sheath can move inside renal pelvis helps to avoid its blockage by ureteral mucosa and AEs related to changes in intra-renal pressure because of this obstruction (24&25).

Additionally, the suction part in novel-UAS helps maintaining diminished level of intra-renal pressure with continuous outflow of irrigation fluid, herby, reducing the incidence of postoperative fever, sepsis and other related AEs [22, 23, 24]).

Qian and co-authors 2022 [25] concurred with existing literature, affirming that the employment of a novel suctioning UAS is associated with a reduced incidence of postoperative fever and systemic inflammatory response syndrome [25].

Gauhar and co-authors 2025 [14] consistently reported in their multicenter investigation that the employment of a FANS during bilateral simultaneous f-URS was associated with a zero incidence of postoperative sepsis and generally low pain scores. Nonetheless, 16.5% of cases developed a postoperative fever exceeding 38.5 °C on the first day following surgery, the majority of whom responded favorably to a single dose of antibiotics and recovered within 24 h. Notably, only four cases exhibited febrile symptoms persisting beyond 48 h. The authors suggested that the elevated rate of fever could be explained by multiple contributing factors, such as a higher stone burden (including cases with stones larger than 2 cm and multiple calculi), positive preoperative urine cultures, the presence of indwelling ureteral stents prior to surgery, utilization of the UAS, and prolonged operative durations [14].

We agree with them on this point, as our univariate binary logistic regression analysis revealed that stone size, pre-operative positive culture, the approach used, and operative time were significant predictors of the risk of post-operative infection.

No doubt that performing bilateral simultaneous flexible URS in general is associated with longer operative time than unilateral cases, but in our study, we exhibited that the employment of FANS decreases the operative time to an acceptable range as there was highly significant increase in operative time in TUAS group versus FANS sheath group (58.4 ± 14.53 in TUAS group versus 48.41 ± 13.9 in FANS group, *p* = 0.001*).

This fact aligns with the results of Gauhar and co-authors 2025 [14] who documented an average operative time of 78 min in their multicenter study on the employment of the FANS for bilateral same-session flexible URS. This duration is well within the recommended safe operative time of 90 min for flexible URS procedures [26].

In alignment with our findings, Zhang and co-authors 2023 [8] also reported a notably reduced surgical duration in cases treated with the FANS in contrast with those managed with the TUAS. These results are consistent with the earlier observations of Zhu and co-authors 2018 ( 21), who documented a statistically significant difference in operative times between the FANS and TUAS groups—reporting mean durations of 49.7 ± 16.3 min versus 57.0 ± 14.0 min, respectively (*P* < 0.001). Similarly, Yu and co-authors (2024) [18] reinforced this trend, demonstrating a significantly shorter operative time in the FANS group when as opposed to the TUAS one, with operative durations of 56.5 min versus 59.9 min, respectively (*P* = 0.047).

Qian and co-authors 2022 [24] conducted a retrospective study to compare between FANS and TUAS groups and they reported a similar difference in terms of mean operative time with 80 min in novel-UAS group versus 72.9 min in TUAS group.

The reduced operative time in the FANS group can be explained by more clear vision, the suction part that allows rapid clearance of stone fragments and decreased need for repeated use of baskets or forceps [25].

In the context of our research findings, a statistically significant difference was observed between the two groups regarding the rate of post-operative ureteral stent placement. Postoperative stenting in our study was done in case of ureteral trauma and residual stones. 58% of cases in the TUAS group required post-operative stenting, as opposed to only 34% in the FANS group, a difference that reached statistical significance (*p* = 0.016*). Conversely, when evaluating the necessity for pre-operative stenting, no statistically significant difference was identified between the two groups, indicating similar baseline characteristics in this regard.

We believe that decreased need for postoperative stenting in FANS group can be explained by its tip characters and its added suction part that helps more efficient and rapid clearance of stones, decreasing incidence of ureteral injury and residual stone fragments, therefore decreasing the need for postoperative stenting.

In view of the above, we believe that Flexible and Navigable Suction Ureteral Access Sheath, despite not being the only one reason to limit simultaneous bilateral surgery, has advantages over traditional UAS in terms of its tip characters and added suction part that improves outcomes with significantly higher SFR, shorter operative times, and a reduced infectious and other ureteral complications and makes surgeon more confident about the decision of same session bilateral flexible ureteroscopy whenever indications of same session bilateral flexible URS are fulfilled.

Our study limitations include, the absence of a device assisted measuring of intra-renal pressure to better validate the theory of decreased intra-renal pressure in FANS group, more multi-center investigations with larger sample size are needed to better evaluate both safety and efficacy of bilateral simultaneous FANS flexible URS and more studies are needed to compare between various sizes of TUAS versus FANS.

## Conclusion

The Flexible and Navigable Suction Ureteral Access Sheath in bilateral simultaneous flexible ureteroscopy emerges as a highly safe and efficient approach, delivering superior outcomes. It boasts a significantly higher SFR, shorter operative times, and a reduced infectious AEs incidence when as opposed to the TUAS, making it a promising advancement in the field.

## Data Availability

Sequence data that support the findings of this study are available upon request.
